# Online medication purchasing during the Covid-19 pandemic: potential risks to patient safety and the urgent need to develop more rigorous controls for purchasing online medications, a pilot study from the United Arab Emirates

**DOI:** 10.1186/s40545-021-00320-z

**Published:** 2021-04-30

**Authors:** Ammar Abdulrahman Jairoun, Sabaa Saleh Al-Hemyari, Naseem Mohammed Abdulla, Faris El-Dahiyat, Maimona Jairoun, Saleh Karamah AL-Tamimi, Zaheer-Ud-Din Babar

**Affiliations:** 1 Health and Safety Department, Dubai Municipality, Dubai, UAE; 2 Department of Pharmacy, Ministry of Health and Prevention, Dubai, UAE; 3 College of Pharmacy, Al Ain University, Al Ain, UAE; 4 College of Pharmacy and Health Sciences, Ajman University, Ajman, UAE; 5 Faculty of Pharmacy, Aden University, Aden, Yemen; 6 Department of Pharmacy, School of Applied Sciences, University of Huddersfield, Huddersfield, HD1 3DH West Yorkshire UK

**Keywords:** Online pharmacies, COVID-19, Patient safety, Online medication

## Abstract

**Background:**

Since the WHO announced that Covid-19 had become a global pandemic, online pharmacies have emerged as an extremely popular way to purchase medication due to the quarantine measures introduced by numerous countries to prevent the virus's spread.

**Aim:**

The aim of this study was to collect information regarding the extent of online medication purchasing in the UAE and to assess the factors that motivating the purchase of medications from the internet.

**Method:**

A convenience sampling of people living in the UAE was used to conduct an online descriptive cross-sectional study. Respondents were solicited using the social media platforms WhatsApp and Facebook, whereby they were asked to fill in a validated web-based questionnaire. The number of people buying medications from online pharmacies was calculated using a percentage with 95% CIs.

**Results:**

131 respondents (31.2%) [95% CI: 26.7–35.6] stated that they purchased medication via the Internet after Covid-19 was classed as a pandemic. It was found that those respondents most likely to have purchased medication via the Internet were male, single, and older and with a high school education.

**Conclusion:**

More research should be conducted to investigate and compare the self-medication and associated risk factors between online pharmacies and community pharmacies. Moreover, regulatory bodies need to make and implement changes to the regulations that govern the sale and use of medications during COVID-19.

## Introduction

Since the start of the Covid-19 pandemic, online pharmacies (OPs) have become a serious public health concern as they have expanded and become more controversial. In brief, OPs are websites that offer to sell medication directly to customers via the Internet [1, 2]. Surveys of other populations have shown that 71% of European Internet users and 72% of US Internet users have run searches for information about health matters at least once in the past year [3–5]. The spread of mobile devices has helped to fuel this boom [6]. Increasingly, consumers are using the Internet to not only find out information about their own health but also to carry out self-diagnosis and purchase health-related products and services, where reliability of information is not trusted due to changes in people’s behaviours and responses due to disease outbreaks especially that the COVID-19 pandemic has been causing a global panic being a health shock [7–12].

In theory, the country in which an OP website is based has control over the licensing and quality control of the OP, and their mail-order business should be undertaken with reference to these standards. However, numerous OPs take pains to keep their physical location concealed, and so there is no certainty as to which, if any, system of regulations any given OP is following [13]. An additional complication is the fact that the power of national regulatory bodies generally ceases at their countries’ borders [14, 15]. This leads to considerable confusion regarding OPs, particularly as the owners of websites selling health services and medication frequently shift their theoretical base between national jurisdictions. This means that research must be done into the home country of an OP and the country from which the purchases are delivered [13]. A number of risks to patient safety exist when purchasing medication online without the traditional supply network, including the risk of receiving falsified medication [16–18].

As Internet pharmacies vary in quality, the legislation appears to be lagging behind in terms of the regulation of this complicated and fast-moving market as regulation of various private drug sellers is still a global challenge contributing to inappropriate treatments particularly at a time when health regulators globally are emphasizing that the population should quarantine themselves at home to restrict the spread of Covid-19 [19]

This research is intended to gather information and discover the extent to which consumers in the UAE are using the Internet for the purchase of medication and to assess the factors that motivating the purchase of medications from the internet. This research may be helpful in developing interventions focused on patients through the identification of consumer patterns.

## Materials and methodology

This research was undertaken using an online descriptive cross-sectional questionnaire deployed between February and April 2020. 420 individuals were issued an invitation through WhatsApp and Facebook to fill in a validated-web-based questionnaire. The sample cohort comprised any individuals over 18 years of age who expressed a willingness to complete the survey. No individuals below the age of 18 were included in the study, nor were any who were unwilling to participate. Recruitment of participants was undertaken using a convenience sampling technique with no pre-determined sampling frame; the researchers asked all their contacts on social networks if they would consider completing the questionnaire and if they would also pass the invitation on to their own social media contacts. The study questionnaire was reviewed and assessed by subject experts to ensure the content validity and reliability of the questionnaire. Additionally, public consultations formed part of the process. The survey was designed such that it could be self-administered. The initial page comprised an introductory paragraph introducing the purpose of the research, confidentiality information, assurance of anonymity, and a series of questions about the demographics of the respondent (age, sex, marital status, level of education, employment, and any chronic disease). The respondents’ patterns of purchasing online medication since the start of the Covid-19 pandemic were revealed through three questions:

- Have you purchased medication from the Internet since the Covid-19 pandemic began? (Yes/No)
- What is the frequency of your purchasing medication via the Internet? (Once or twice/several times/Frequently)
- What types of medicine have you purchased from the Internet?

Any participant who continued to the following page was regarded as having consented to be a participant in the study. No participant identities were recorded and confidentiality was guaranteed. When the survey was completed, all respondents received a thank you message from the researchers. No form of incentive was offered to complete the survey. Data analysis was undertaken using SPSS version 24. The number of individuals making purchases from the internet was rendered as a percentage (95% CI). Factors motivating the purchase of medications from the internet were assessed using simple binary logistic regression in which a *p*-value < 0.05 was regarded as statistically significant.

## Results

### Sociodemographic characteristics of the study participants

A total number of 420 respondents participated in the study. Of the total participants, 195 (46.4%) were female and 225 (53.6%) were male. The ages of the 420 participants were distributed as follows: 50 (11.9%) were aged 18–24, 97 (23.1%) were aged 25–34, 85 (20.2%) were aged 35–44, 129 (30.7%) were aged 45–54, and 59 (14%) were aged ≥ 55. Most of the participants were married (*n* = 322, or 76.7%). The educational qualifications of the participants also varied: 12 (2.9%) were high school education holders, 140 (33.3%) held bachelor’s degrees, and 268 (63.8%) had attained post-graduate education. Of the total number of participants, 308 (73.3%) were employed and 332 (79%) did not have a chronic disease (Table 1).

**Table 1:** Number and percentages of the sociodemographic characteristics (*n* = 420)

Demographic factors	Groups	Frequency	Percentages
Sex	Female	195	46.4%
	Male	225	53.6%
Age groups	18–24	50	11.9%
	25–34	97	23.1%
	35–44	85	20.2%
	45–54	129	30.7%
	≥ 55	59	14%
Marital status	Single	98	23.3%
	Married	322	76.7%
Education level	High school	12	2.9
	Bachelor’s degree	140	33.3%
	Postgraduate	268	63.8%
Employment status	Unemployed	112	26.7%
	Employed	308	73.3%
Chronic disease	Yes	88	21%
	No	332	79%

### Prevalence of online medication purchasing during the Covid-19 pandemic

In this study, 131 participants (31.2%) [95% CI: 26.7–35.6] reported having purchased medication from the Internet during the Covid-19 pandemic. Of these 131 participants, 118 (91.1%) had purchased several times, seven participants (5.3%) had purchased frequently, and six participants (4.6%) had purchased once or twice.

The categories of medication purchased online during the pandemic were as follows: 26 (19.8%) participants had purchased analgesics, 14 (10.7%) participants had purchased anti-cough medicine, 66 (50.4%) participants had purchased dietary supplements, 10 (7.6%) had purchased nasal sprays, and 15 (11.5%) had purchased antihistamines. The ingredients of each medication group are shown in Table 2.

**Table 2:** Categories of medications purchased from OPs

Medication category	Frequency	Percentage	Medication name
Analgesic	26	19.8%	Panadol (Panadol cold and flu) Brufen (ibuprofen) Voltfast (Diclofenac potassium) Aspirin + vit C Ponstan (Mefenamic acid)
Anti-cough medicine	14	10.7%	Prospan Propolsaft Amydramin Mucosulvan Toplexil Zecuf
Dietary supplements	66	50.4%	Manuka honey Royal jelly Propolis Echinacea Vitamin C Zinc Vitamin D Immune booster Velvet power Black elderberry Multivitamins & mineral s
Nasal spray	10	7.6%	Otrivin spray Betadine Cold Defence Betadine Soothing Relief Normal saline Normal saline + propolis Normal saline + copper
Antihistamine	15	11.5%	Telfast (Fexofenadine HCL) Aerius (Desloratadine) Claritine (Loratadine)

In the present study, a significantly increased prevalence of purchasing medication online was observed in male subjects [OR 1.71; 95% CI 1.12–2.61], single marital status participants [OR 2.08; 95% CI 1.30–3.32], and high school education holders [OR 6.93; 95% CI 1.83–26.3]. However, purchasing medication online was less prevalent in participants aged 35–44 [OR 0.36; 95% CI 0.18–0.76] and aged 45–54 [OR 0.41; 95% CI 0.21–0.79] (see Table 3).

**Table 3:** Prevalence of online medication purchasing according to sociodemographic characteristics (*n* = 420)

		Prevalence of OPs				
Variable	Groups	Estimate	OR	95% CI		*P*-value
				Lower	Upper	
	**All**	131 (31.2%)		26.7	35.6	
Sex	Female	49 (25.1%)	**1**	–	–	–
	Male	82 (36.4%)	1.71	1.12	2.61	0.013
Age groups	18–24	20 (40.0%)	0.91	0.42	1.95	0.80
	25–34	38 (39.2%)	0.88	0.45	1.69	0.63
	35–44	18 (21.2%)	0.36	0.18	0.76	0.007
	45–54	30 (23.3%)	0.41	0.21	0.79	0.008
	≥ 55	25 (42.4%)	**1**	–	–	–
Marital status	Single	43 (43.9%)	2.08	1.30	3.32	0.002
	Married	88 (27.3%)	**1**	–	–	–
Educational level	High school	9 (75.0%)	6.93	1.83	26.3	0.004
	Bachelor’s degree	41 (29.3%)	0.96	0.61	1.49	0.84
	Postgraduate	81 (30.2%)	**1**	–	–	–
Employment status	Unemployed	34 (30.4%)	**1**	–	–	–
	Employed	97 (31.5%)	1.06	0.66	1.69	0.82
Chronic disease	Yes	107 (30.8%)	0.97	0.58	1.61	0.91
	No	24 (32.9%)	**1**	–	–	–

P-values less than 0.05 were considered statistically significant

*OR* odds ratio, *CI* confidence interval

## Discussion

Since the WHO classed Covid-19 as a global pandemic, OPs have become an increasingly frequent means of purchasing medication as a result of the quarantine conditions that numerous nations have introduced in order to mitigate the spread of the virus. Recent investigations have suggested that the number of people using OPs to obtain medication and other health products is on the increase [20]. A number of surveys have shown that the proportion of people using OPs fluctuates depending on the survey focus and is influenced by product type, income, population, education, and/or problems with substance abuse [1, 21]. This study looked at the proportion of people using the Internet to purchase medication since the start of the Covid-19 pandemic. One-third of respondents to the survey (31.2%) stated that they had made medication purchases from the Internet due to the pandemic. The most frequently purchased medication from the internet were dietary supplements (50.4%), analgesics (19.8%), antihistamines (11.5%), and anti-cough medicine (10.7%), It should be noted that no research is currently available regarding the percentages of drug purchases from the Internet during the Covid-19 pandemic, but past research can be used to examine variations in the pattern of purchases. Research in Saudi Arabia demonstrated that online medication purchases were not commonplace at the time of the research, with only 23.1% of respondents stating that they knew about the availability of OPs, and only 2.7% reporting having actually made medication purchases online [22]. Alfahad et al. [23] returned similar findings: 82.6% of their survey sample in Saudi Arabia knew nothing about OPs, and only 1.4% of respondents had used them to buy medication.

The outcomes of this research demonstrate that the respondents most likely to make online purchases of medication were unmarried, male, older, and holders of a high school education.

In a survey conducted in the USA on 443 customers of OPs found that in comparison to those who did not use such pharmacies, users were of a higher age bracket on average, had higher family incomes, a higher level of education, and a greater number of prescriptions and there was a greater likelihood of them having private medical insurance [24]. Atkinson et al. [25] revealed significant associations between age/marital status and the online purchase of medication. Nevertheless, gender, age, and/or education level did not appear to have a significant influence on the purchase of medication or health products online [26].

There are a number of patient safety issues in relation to the purchase of medications via the Internet during the Covid-19 pandemic. Those who use OPs, legitimate or illegitimate, purchase medications for chronic and acute conditions, and these may include medications that can be abused. Any drug, even dietary supplements, may be harmful if administered without advice and/or supervision from a physician or pharmacist. The chief concerns regarding the purchase of online medication are that OPs do not take a rigorous history of patient conditions and medications, patients may self-diagnose inappropriately, there may be clashes between medications, patients may undergo more than one type of therapy at once, and there may be reactions between different drugs and/or drugs and herbal remedies [27]. Clearly, illegitimate OPs are a significant patient threat when they sell sub-standard medication or poor quality products, but even legitimate pharmacies have the potential to cause serious adverse health outcomes for users [20].

In this time of crisis, it is crucial that OPs be more robustly controlled and that consumers be educated regarding the ways in which they purchase medication online. Past research has shown that more emphasis should be placed on the way in which healthcare providers (physicians, pharmacists, and nurses) can assist patients by discussing with them their intentions in terms of online medication purchasing; this could greatly reduce the number of adverse outcomes related to purchasing medication from unlicensed or illegitimate online retailers [20, 26].

The key differences in the online medication purchasing behaviors before and during Covid-19 crisis is the excessive and unregulated use of the medication. As reported in this study, older participants and higher school education holders more likely to practice online medication purchasing and this segment of society may self-medicated and misuse these medicines as they may suffer from chronic diseases and do not have sufficient knowledge to use the medicines, especially since these medicines are taken without consulting a doctor and pharmacist.

Moreover, the enormous stress of the Covid-19 pandemic directed the mentality of many people of different backgrounds and diversities towards the idea of enhancing their immunity to be able to fight the Covid-19 virus in case of getting infected. The first weapon most people think of to do that is starting to take different types of vitamins and herbal supplements. For instance, many people started to purchase multivitamins, vitamin D supplements, Vitamin C, Echinacea products, garlic and ginger capsules.

Such practice is done mainly through net surfing, as this method is the most convenient one in order to stay away from the streets and the direct contacts with others. This way they would feel safer, and whatever products they think would help them get healthier would reach them at their doors.

In addition to that, melatonin, and sleep aids were being sold out significantly as many people needed something to help them sleep and relax under all the stress they are facing.

Another concern regarding the online self-medication practice during Covid-19 pandemic concerns questions surrounding the purity, quality, and authenticity of the medications that are available. Lately, a safety study was held in Dubai, UAE, by which 6/102 tested alcohol-based skin sanitizers revealed to contain unlisted or undeclared methanol encompassed with the ingredients. Moreover, others revealed containing alcohol of less than 60% although their labels stated to contain alcohol of 70% [28]. A relatively similar survey in Ajman, UAE, lead to shutting two factories down after a great amount of sub-standard sterilizer was captured—an approximate amount of 40,000 pieces holding a market value of about 500,000 United Arab dirhams. When the placed stickers were removed from the products, the original below sticker unveiled that they were nothing but body spray perfumes, not at all medically sterile products as per pretended [29]. Such unaccepted practices rings the bell for medication safety attention.

Further, significant concern during Covid-19 pandemic is that numerous non-specialist manufacturers began reorienting their activities towards the production of different medications, dietary supplements, cosmetics and personal care products. As a result, many unregulated medications and supplements have entered the market. Many of these online medications and supplements claimed to be effective against Corona virus disease without documented evidential support. Hence, public can be misled by such advertising claims on the Internet, which cannot be verified by competent authorities raising the concern of medication safety.

Therefore, health and regulatory bodies need to make and implement changes to the Implementing Regulations that govern the use and sale of medications during COVID-19. In addition, stricter enforcement measures and tests need to be established to ensure that medications that are sold online are safe, of a sufficient quality, and authentic.

To more strictly control the sale of medication online, online providers should be required to register their business and publish all safety-related information within a central, government regulated database. Customers can subsequently access this system to verify suppliers and report and safety concerns or violations. There is also a requirement to raise general awareness of the need to verify the safety of medication products that are bought online.

It is strongly suggested that future studies should highlight the adverse effects that arise from medications being purchased through internet. New evolving technologies, like “machine learning algorithms” that are used in application of “Big Data,” is the platform for an emerging area of medical research named as “digital” research or “infoveillance. In accordance to this, risk of patient safety in outpatient settings can be measured by different methods. Enhanced patient-healthcare professional education, communication and promotion campaigns are required to educate the public regarding the safe approach of online pharmacies. Targeted interventions by pharmacists are vital as prevention strategies that should be stressed in the daily practice.

There are certain limitations to this study. The use of OPs was self-reported and so could be warped by recall bias and/or false reporting from the respondents, possibly leading to an underestimation of the true figures. Furthermore, this research does not differentiate between legitimate and illegitimate retailers, chiefly because all online sellers of medication pose as legitimate, so respondents would be unlikely to know with certainty what their status was.

## Conclusion

This research demonstrated that around one-third of those responding had used the Internet to purchase medication during the Covid-19 pandemic. Large-scale longitudinal research should be undertaken to gain further details regarding the purchase of medication online. Additionally, more research should be conducted to investigate and compare the self-medication and associated risk factors between online pharmacies and community pharmacies.

## Data Availability

The datasets generated during and/or analysed during the current study are available from the corresponding author on reasonable request.
