# Correction to: Online medication purchasing during the Covid-19 pandemic: a pilot study from the United Arab Emirates

**DOI:** 10.1186/s40545-021-00324-9

**Published:** 2021-05-11

**Authors:** Ammar Abdulrahman Jairoun, Sabaa Saleh Al‑Hemyari, Naseem Mohammed Abdulla, Faris El‑Dahiyat, Maimona Jairoun, Saleh Karamah AL‑Tamimi, Zaheer‑Ud‑Din Babar

**Affiliations:** 1 Health and Safety Department, Dubai Municipality, Dubai, UAE; 2 Department of Pharmacy, Ministry of Health and Prevention, Dubai, UAE; 3 College of Pharmacy, Al Ain University, Al Ain, UAE; 4 College of Pharmacy and Health Sciences, Ajman University, Ajman, UAE; 5 Faculty of Pharmacy, Aden University, Aden, Yemen; 6 Department of Pharmacy, School of Applied Sciences, University of Huddersfield, Huddersfield, HD1 3DH West Yorkshire UK

## Correction to: J of Pharm Policy and Pract (2021) 14:38 https://doi.org/10.1186/s40545-021-00320-z

Following the publication of the original article [1], the authors notified us of a few errors, reflected below:

1. Title:
  Originally published: “Online medication purchasing during the Covid-19 pandemic: potential risks to patient safety and the urgent need to develop more rigorous controls for purchasing online medications, a pilot study from the United Arab Emirates”.
  Corrected: “Online medication purchasing during the Covid-19 pandemic: A pilot study from the United Arab Emirates”.

2. Reference 9
  Original (incomplete): “Bukhari N, Rasheed H, Nayyer B, Babar ZUD (2020) Pharmacists at the ‏frontline beating the COVID‐19 pandemic”.
  Updated: “Bukhari, N., Rasheed, H., Nayyer, B. et al. Pharmacists at the frontline beating the COVID-19 pandemic. J of Pharm Policy and Pract 13, 8 (2020). https://doi.org/10.1186/s40545-020-00210-w”.

The original article has been corrected.
